# Dietary Rutin Ameliorates Nanoparticle Zinc Oxide-Induced Toxicity in Mice by Potentiating Antioxidant Defense Mechanisms

**DOI:** 10.3390/nu17091495

**Published:** 2025-04-29

**Authors:** Xiaofang He, Longfei Ma, Jiaqi Zhang, Binbin Zhou, Shun Chen, Minhang Tu, Gentan Cai, Tian Wang, Chao Wang

**Affiliations:** 1 College of Animal Science and Food Engineering, Jinling Institute of Science and Technology, Nanjing 211169, China; hexiaofang@jit.edu.cn; 2 College of Animal Science and Technology, Nanjing Agricultural University, Nanjing 210095, China; 2022105076@stu.njau.edu.cn (L.M.); 2022205046@stu.njau.edu.cn (J.Z.); 2020105063@stu.njau.edu.cn (B.Z.); 2021105066@stu.njau.edu.cn (S.C.); 2023105076@stu.njau.edu.cn (M.T.); 2023805148@stu.njau.edu.cn (G.C.); tianwang@njau.edu.cn (T.W.)

**Keywords:** zinc oxide nanoparticle, rutin, hepatic function, mitochondrial dysfunction, antioxidant capacity

## Abstract

In animal production, nanoparticulate zinc oxide exhibits synergistic antibacterial efficacy coupled with growth-promoting effects, positioning itself as a novel antibiotic alternative with enhanced biosafety profiles. However, its dose-dependent toxicity poses challenges. **Objective:** The experimental design sought to quantify the protective effects of dietary rutin against zinc-overload-induced damage. **Methods:** A zinc-overload murine model was established by giving high-dose ZnO nanoparticles (HZn, 5000 mg/kg/day) for 21 days. Mice were then fed rutin at doses of 300, 600, or 1200 mg/kg. Body weight, relative organ indexes, zinc concentrations, serum enzyme activities, and tissue-level indicators of apoptosis, autophagy, mitochondrial function, and antioxidant capacity were measured. **Results:** The results showed that rutin could not reverse HZn-induced body weight decline but improved relative organ indexes in liver and kidney. It alleviated HZn-induced cell damage and enhanced antioxidant capacity in jejunum and serum through *Nrf2* activation, without inhibiting HZn-induced zinc elevation. **Conclusions:** Rutin, especially at 600 mg/kg, can partially restore hepatic function and organ index and mitigate HZn-induced hepatic and jejunal injuries.

## 1. Introduction

Nanoparticulate zinc oxide (nano-ZnO) is an attractive material in many fields for its multifunctional properties and easy synthesis [1,2,3]. However, nano-ZnO poses underlying threats to people’s safety due to its potential negative effects [4,5]. Studies found that long-term dietary supplementation with 5000 mg/kg nano-ZnO caused the inhibited development of mice and altered the zinc metabolism and biodistribution [6]. Moreover, gastrointestinal administration of 250 mg/kg nano-ZnO in mice for 7 weeks can also reduce body weight and exhibit hepatic toxicity [7]. Yan et al. [8] found that nano-ZnO could disturb the energy metabolism in the rat kidney and cause mitochondrial membrane impairment. These findings suggest that high-dose nano-ZnO (HZn), especially for long-term use, could show toxicity to organ development and function, such as hepatic and intestinal functions. The main mechanism can be correlated with the high-zinc-induced oxidative stress in tissues, which could further cause lethal cellular damage and activate the apoptotic signaling pathway [9,10,11]. Accumulating evidence indicates that antioxidants, especially the natural extracts, could attenuate or even effectively reverse the possible toxicity induced by high-dose metals via improving antioxidant capacity or mitochondrial function [12]. Therefore, dietary natural antioxidants might be an effective and safe way to reduce nano-ZnO toxicity in animals.

Rutin (quercetin-3-*O*-rutinoside), a glycosylated flavonol derivative, is ubiquitously distributed in plant-based dietary sources including citrus fruits (oranges, limes), solanaceous vegetables (tomatoes), vitaceous berries (grapes), and herbal infusions, with particularly high concentrations observed in buckwheat and apple peels [13,14]. Rutin contains phenolic hydroxyl groups as a strong antioxidant [13,15]. Studies have reported that gavage of 70 mg/kg rutin in male rats attenuated CCl4-induced hepatotoxicity, nephrotoxicity, and reproductive toxicity [16]. Gautam et al. [17] found that 50 and 100 mg/kg rutin showed significant protection against methotrexate-induced intestinal toxicity. These studies suggest that dietary supplementation with rutin, a plant extract with strong antioxidant capacity, might mitigate the toxicity induced by HZn. Therefore, the growth, antioxidant capacity, intestinal function, and zinc distribution in mice were determined to explore the potential role of rutin in mitigating negative effects of HZn.

## 2. Materials and Methods

Experiments were approved and conducted under the supervision of the Institutional Animal Care and Use Committee of Nanjing Agriculture University (Nanjing, China).

### 2.1. Animal Treatment, Experimental Design, and Sample Collection

A total of 40 Improved Castle Road (ICR) male mice (21 d) were randomly divided into 5 groups, each group with 8 mice. In this study, group allocation was performed by a researcher using a randomized block design, with cages labeled numerically to blind animal caretakers during daily monitoring. Personnel administering nano-ZnO and rutin-supplemented diets were aware of group assignments to ensure treatment accuracy. Based on our previous study [6], these mice were fed diets as follows for 21 days. The experimental groups were designed as follows:

(1) Control group: Fed a basal diet;
(2) HZn group: Fed a basal diet supplemented with 5000 mg/kg nano-ZnO;
(3) HZn + R1 group: Fed a basal diet supplemented with 5000 mg/kg nano-ZnO and 300 mg/kg rutin;
(4) HZn + R2 group: Fed a basal diet supplemented with 5000 mg/kg nano-ZnO and 600 mg/kg rutin;
(5) HZn + R3 group: Fed a basal diet supplemented with 5000 mg/kg nano-ZnO and 1200 mg/kg rutin.

Nano-ZnO was provided by Zhangjiagang Bonded Area Hualu Nanometer Material Co., Ltd. (Suzhou, China), and rutin was purchased from Shanghai Aladdin Biotechnology Co., Ltd. (Shanghai, China). During this feeding trial, all mice were housed in polypropylene cages at room temperature (22 ± 2 °C) with a 12 h light/dark cycle. Mice were fed with deionized water and ad libitum and monitored for general appearance and status daily. At 0, 7, 14, and 21 d of this feeding trial, all mice were weighed individually. After the feeding trial and fasting for 12 h, blood samples were collected from the orbital venous plexus of anesthetized mice, and then the obtained blood samples were centrifuged at 3500× *g* for 10 min to obtain serum samples. The livers, kidneys, spleens, and testes were collected and weighed to calculate the relative organ index (organ mass (g)/body mass (kg). The left liver (1 cm × 1 cm) samples were fixed in 4% formaldehyde, and the jejunum (1.5 mm × 3 mm) were fixed in 2.5% buffered glutaraldehyde for further assays. The tibia and remaining liver samples were stored at −20 °C, and the remaining jejunum samples were frozen at −80 °C rapidly for subsequent analysis.

### 2.2. Analysis of Nano-ZnO Charaterization

The characterization of nano-ZnO was analyzed according to our previous method [18]. Briefly, nano-ZnOs were firstly placed on a carbon-coated copper grid, and then transmission electron microscope (TEM, Hitachi H-7650, Tokyo, Japan) with a Hitachi TEM system was used to observe the primary particle size and morphological characterization of nano-ZnO at an accelerating voltage of 80 kV.

### 2.3. Analysis of Serum and Jejunal Parameters

The serum and jejunal malondialdehyde (MDA, A003-1-1) content, total superoxide dismutase (T-SOD, A001-1-1) activities in serum and jejunum, serum activities of glutamic oxalacetic transaminase (GOT, C010-1-1), glutamic pyruvic transaminase (GPT, C009-1-1), alkaline phosphatase (AKP, A059-2-2), and jejunal total antioxidant capacity (T-AOC, A015-1-2) were measured using corresponding commercial kits (Nanjing Jiancheng Bioengineering Institute, Nanjing, China) following the manufacturer’s recommended protocols.

### 2.4. Histological Analysis

The fixed jejunum samples (2.5% buffered glutaraldehyde) were cleaned, further fixed (1% osmium tetroxide), dehydrated (50%, 70%, 80%, 90%, and 100% acetone), embedded, and cut to obtain the ultrathin sections (stained with 3% uranyl acetate solution and 3% lead citrate solution), which were further observed under the TEM system (Hitachi H-7650, Tokyo, Japan) at an accelerating voltage of 80 kV.

The liver samples fixed in 4% buffered formaldehyde were dehydrated through a graded series of xylene and ethanol, impregnated with paraffin wax, and then sectioned into 5 μm slices. The sections were deparaffinized and stained with hematoxylin and eosin (H&E). Images were captured using a Nikon H550L microscope (Tokyo, Japan) equipped with a digital camera.

### 2.5. Determination of Zinc Concentration

The zinc concentrations of serum, liver, and tibia were determined as in our previous study [19]. Briefly, the samples (0.5–1.0 g) were digested using an acid mixture (HNO_3_:HClO_4_ = 4:1, *v*:*v*) and diluted with deionized water to 25 mL. After standard curves were determined with Zn standard, zinc content was analyzed with inductively coupled plasma optical emission spectrometry (Palo Alto, CA, USA).

### 2.6. Analysis of mRNA Expression

In this study, the previous methods [19,20] were employed to assess the mRNA expression of genes including BCL2-associated X protein (Bax), caspase-3, Ki67, P53, heat shock protein 90 (Hsp90), heat shock protein 40 (Hsp40), glutathione peroxidase-1 (Gpx1), nuclear factor erythroid-2-related factor 2 (Nrf2), superoxide dismutase-1 (Sod1), dynamin 1-like (Dnm1l), mitofusin 2 (Mfn2), fission protein 1 (Fis1), and β-actin. The primers are shown in Table 1. The mRNA expression levels of the target genes were normalized to the housekeeping gene β-actin to calculate the relative mRNA expression using the 2−ΔΔCt method [20].

### 2.7. Statistical Analysis

Statistical analyses were performed using SPSS 26.0 software (SPSS Inc., Chicago, IL, USA). Data were analyzed by one-way analysis of variance (ANOVA) for comparisons across all groups, followed by Tukey’s post hoc test for pairwise comparisons. A value of *p* < 0.05 was considered statistically significant among groups.

## 3. Results

### 3.1. The Characterization of Nano-ZnO

**Figure 1:**
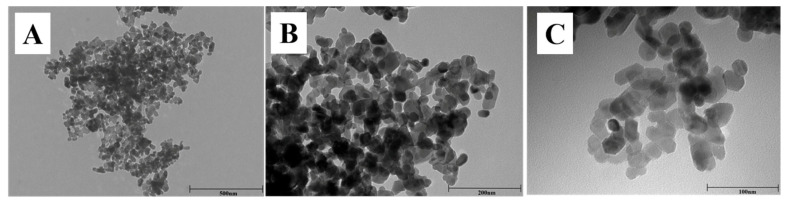
TEM images of nano-ZnO at low (**A**), medium (**B**), and high (**C**) magnifications.

As shown in Figure 1, the TEM images indicate that nano-ZnOs used in our present study showed nearly spherical geometry with diameters about 30 nm (mainly ranging from 20 nm to 40 nm).

### 3.2. Body Weight

**Figure 2:**
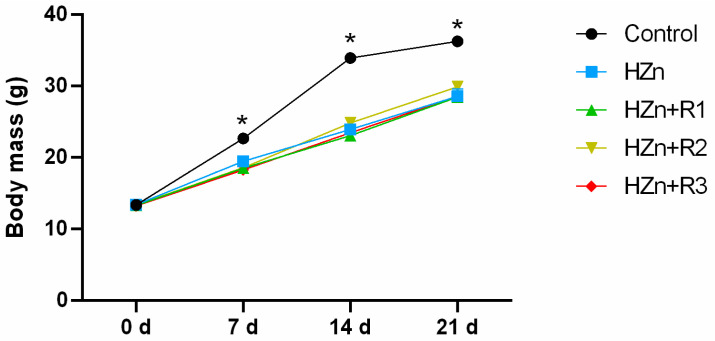
Effects of dietary rutin on body weight in mice treated by high dose of nano-ZnO at 0 d, 7 d, 14 d, and 21 d. * means that the values within that group were significantly different (*p* < 0.05).

As shown in Figure 2, in comparison with the control group, HZn group significantly decreased the body weight at 7 d, 14 d, and 21 d (*p* < 0.05), while there was no significant difference in body weight of mice in the HZn + R1, HZn + R2 and HZn + R3 groups when compared to the HZn group (*p* > 0.05).

### 3.3. The Relative Organ Indexes

**Figure 3:**
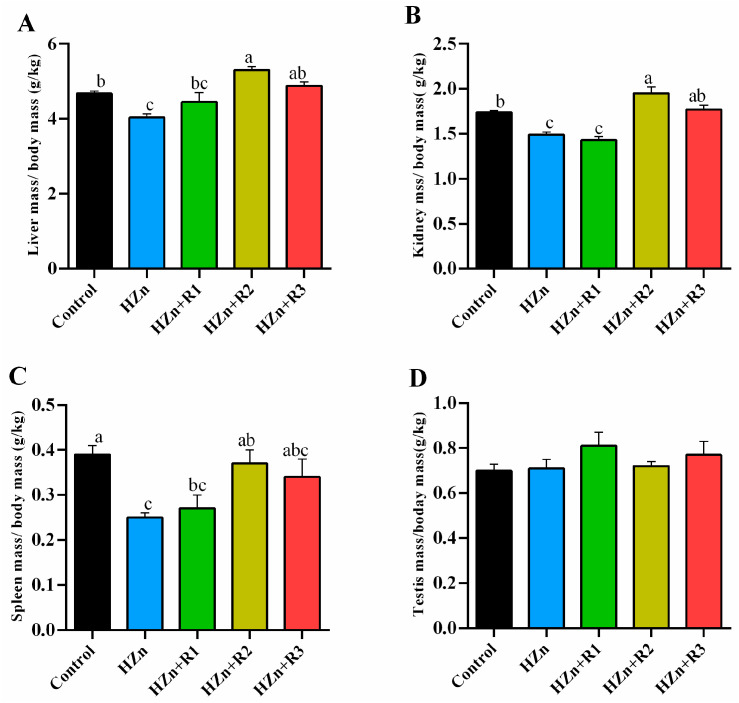
Effects of dietary rutin on relative organ indexes (liver (**A**), kidney (**B**), spleen (**C**), and testis (**D**)) in mice treated with high doses of nano-ZnO. Values with different superscripts (a, b, c) indicate significant differences (*p* < 0.05). Data are presented as mean ± standard error (SE, *n* = 8).

As shown in Figure 3, compared with the control group, the HZn group significantly decreased the relative organ indexes of the liver, kidney, and spleen (*p* < 0.05). Compared with the HZn group, HZn + R2 and HZn + R3 groups significantly increased the relative organ indexes of the liver and kidney (*p* < 0.05), and the HZn + R2 group significantly increased the relative organ index of the spleen (*p* < 0.05). There is no difference in the testes among groups (*p* > 0.05).

### 3.4. Zinc Concentrations in Serum, Liver, and Tibia

**Figure 4:**
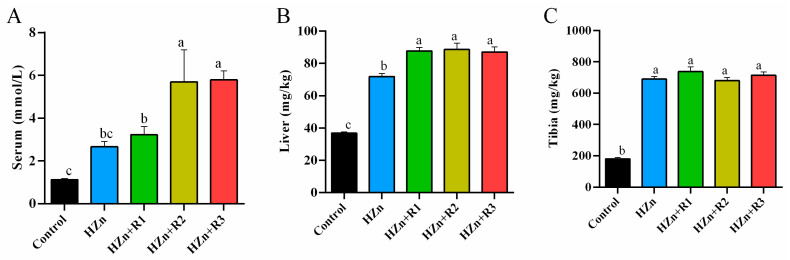
Effects of dietary rutin on Zn concentrations in serum (**A**), liver (**B**), and tibia (**C**) of mice treated with high doses of nano-ZnO. Values with different superscripts (a, b, c) indicate significant differences (*p* < 0.05). Data are presented as mean ± standard error (SE, *n* = 8).

As shown in Figure 4, compared with the control group, mice in the HZn group had higher Zn concentrations in the liver and tibia (*p* < 0.05). Compared with the HZn group, treatment groups supplemented with different concentrations of rutin did not significantly affect the zinc level in the tibia (*p* > 0.05). While HZn + R1 caused a significant rise in the zinc content of the liver, both HZn + R2 and HZn + R3 groups increased the serum Zn concentration (*p* < 0.05).

### 3.5. Histological Analysis of Liver

**Figure 5:**
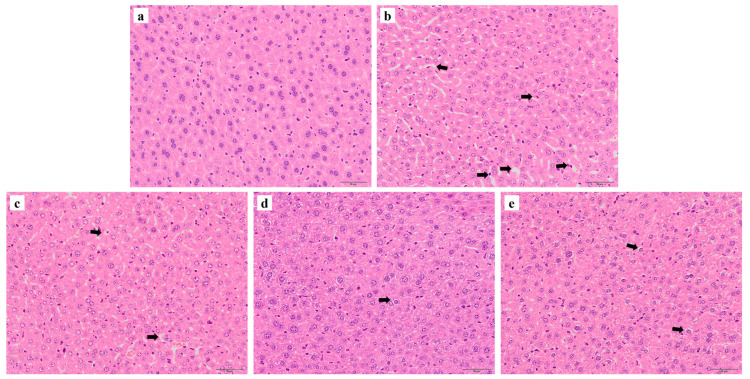
Effects of dietary rutin on hepatic histology in mice treated with high doses of nano-ZnO. Photomicrographs (stained with hematoxylin and eosin) of livers collected from the different groups, including the control (**a**), HZn (**b**), HZn + R1 (**c**), HZn + R2 (**d**), and HZn + R3 (**e**) groups. Arrows indicate that the arrangement of hepatocytes was disordered, and the intercellular space was larger.

As shown in Figure 5, the hepatocyte in the control group exhibited normal architecture with well-preserved cytoplasm, while the cell structure in the HZn group showed obvious dissolution, disarrangement, disorder, and larger intercellular space. Compared to the HZn group, treatment groups supplemented with different concentrations of rutin partially attenuated these histopathological alternations.

### 3.6. Serum Activities of GOT, GPT, and AKP

**Figure 6:**
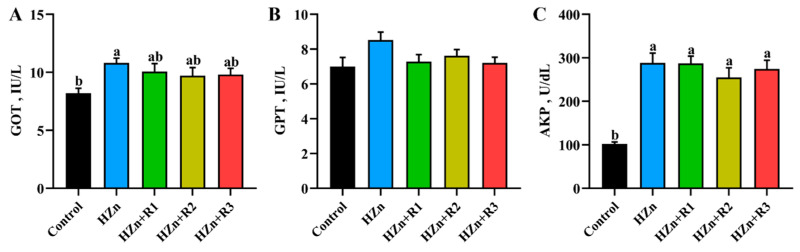
Effects of dietary rutin on serum activities of GOT (**A**), GPT (**B**), and AKP (**C**) in mice treated with high doses of nano-ZnO. Values with different superscripts (a, b) indicate significant differences (*p* < 0.05). Data are presented as mean ± standard error (SE, *n* = 8).

As shown in Figure 6, compared with the control group, dietary HZn did not alter GPT activity (*p* > 0.05) but obviously increased serum activities of GOT and AKP (*p* < 0.05). There were no significant differences in the activities of serum GPT, GOT, and AKP among the HZn group and the three treatment groups supplemented with different concentrations of rutin (*p* > 0.05).

### 3.7. TEM Analysis of the Jejunum Histology

**Figure 7:**
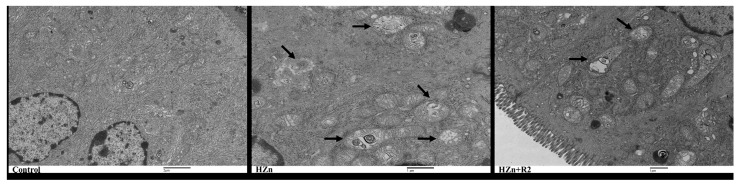
Effects of dietary rutin on jejunum histology in mice treated with high doses of nano-ZnO. Images represented jejunal histology from the control (control: basal diet), HZn (HZn: basal diet + 5000 mg/kg nano-ZnO), and HZn + R2 (HZn + R2: basal diet + 5000 mg/kg nano-ZnO +600 mg/kg rutin) groups, with arrows indicating swollen or vacuolated mitochondria.

As compared to the control group, the HZn group showed obvious mitochondrial vesiculation and autophagy in jejunum, while the HZn + R2 group significantly attenuated the swollen mitochondria and decreased the amount of autophagy (Figure 7).

### 3.8. Serum and Jejunal Redox Status

**Figure 8:**
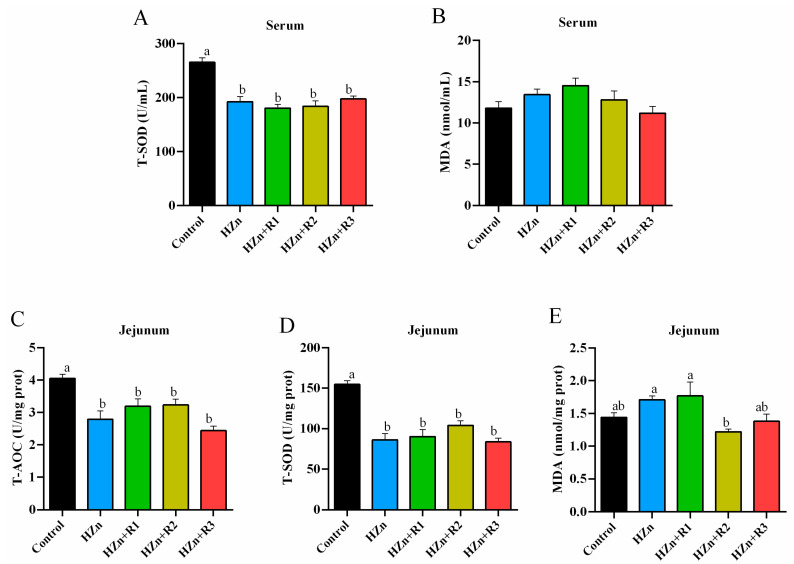
Effects of dietary rutin on antioxidant status in serum (**A**,**B**) and jejunum (**C**–**E**) of mice treated with high doses of nano-ZnO. Values with different superscripts (a, b) indicate significant differences (*p* < 0.05). Data are presented as mean ± standard error (SE, *n* = 8).

As summarized in Figure 8, compared with the control group, dietary HZn significantly (*p* < 0.05) decreased the T-SOD activity (in serum and jejunum) and the T-AOC level (in jejunum), and significantly increased the jejunal MDA content, which was significantly decreased in the HZn + R2 group as compared to the HZn group (*p* < 0.05). There was no significant difference in serum MDA levels among the five groups (*p* > 0.05).

### 3.9. Gene Expression Involved in Antioxidant Capacity, Apoptosis, and Mitochondrial Function in Jejunum

**Figure 9:**
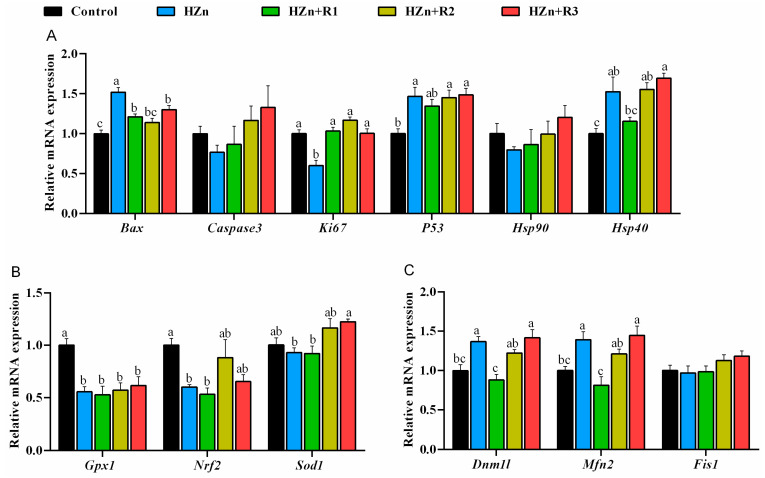
Effects of dietary rutin on mRNA expression involved apoptosis (**A**), antioxidant (**B**), and mitochondrial function (**C**) in jejunum of mice treated with high doses of nano-ZnO. Values with different superscripts (a, b, c) indicate significant differences (*p* < 0.05). Data are presented as mean ± standard error (SE, *n* = 8).

As shown in Figure 9A, dietary HZn significantly upregulated mRNA expression of *Bax*, *Hsp40*, and *P53*, whereas it downregulated the mRNA expression of *Ki67* in jejunum (*p* < 0.05). When compared with the HZn group, treatment groups supplemented with different concentrations of rutin significantly decreased *Bax* and increased *Ki67* mRNA expression (*p* < 0.05), and HZn + R1 significantly decreased *Hsp40* mRNA expression in jejunum (*p* < 0.05). Figure 9B showed that HZn significantly (*p* < 0.05) downregulated the jejunal *Sod1*, *Gpx1*, and *Nrf2* mRNA expression, in which no significant difference was observed among the HZn + R2, HZn + R3, and control groups (*p* > 0.05), whereas HZn + R3 significantly increased the *Sod1* mRNA expression as compared with the HZn group (*p* < 0.05). Figure 9C showed that dietary HZn did not change *Fis1* expression (*p* > 0.05) but significantly upregulated the mRNA expression of *Dnm1l* and *Mfn2* (*p* < 0.05), in which no significant difference was observed between the HZn + R2 and control groups (*p* > 0.05). HZn + R1 significantly downregulated the mRNA expression of *Dnm1l* and *Mfn2* compared to that in the HZn group (*p* < 0.05).

## 4. Discussion

As for its high bioavailability, increased surface area, and small particle size, nano-ZnO exhibits multiple properties and holds great potential in various fields, such as food additives, biomedicine, and agriculture [21]. Nano-ZnO has emerged as a promising alternative to conventional zinc oxide supplements, which are commonly employed in pediatric and animal nutrition formulations. The nanoscale material demonstrates enhanced bioavailability and reduced dosage requirements while maintaining the essential nutritional functions of traditional ZnO additives [2,22]. However, the toxicity of nano-ZnO has been reported in both in vitro and in vivo studies, which may be attributed to the high oxidative stress induced by nano-ZnO. The characterization of nano-ZnO, including the shape and particle size, is one of the most important factors affecting the nano-ZnO-induced cytotoxicity. The spherical nano-ZnO shows higher cytotoxicity than flower-like nanoparticle or nano-ZnO tetrapods, and all these nanoparticles of ZnO exhibit higher toxicity as particle size decreases [23,24,25]. Therefore, the characterization of the nano-ZnO used in this study was determined. Results indicated that these nano-ZnO particles showed nearly spherical geometry with an average particle size of about 30 nm (mainly ranging from 20 nm to 40 nm), which aligns with the previous study conducted by Wang et al. [6]. Moreover, our consequences indicated that the body weight of the HZn group was significantly decreased (7 d, 14 d, and 21 d), as were the relative organ indexes (liver, kidney, and spleen) in weaned mice, which are also consistent with outcomes elucidated by Wang et al. [6]. However, our present study found that dietary rutin, especially for dietary 600 mg/kg, effectively attenuated the HZn-induced decrease in the relative organ indexes (liver, kidney, and spleen). These data align with previous findings demonstrating that rutin protects the liver and kidneys by counteracting the toxicity of synthetic and natural compounds toward these organs [26]. Although in previous reports, dietary rutin showed promoting effects on growth of animals [27,28], in our study, dietary 300–1200 mg/kg rutin had no obvious effects on the inhibited growth induced by HZn in mice, which might be related to the short feeding time or excessive zinc absorbed into the body [6].

Results of the zinc content in serum and tissues indicated that dietary rutin did not attenuate the HZn-induced high zinc concentration in the tibia and even further increased the liver and serum zinc contents by 600 and 1200 mg/kg rutin as compared with the HZn group, which could partially explain why the HZn-inhibited body weight was not improved by dietary rutin [6]. The gastrointestinal tract acts as the primary site for the enzymatic breakdown and transmembrane absorption of nutritional substrates. It coordinates a hierarchical process comprising mechanical fragmentation, luminal hydrolysis, and enterocyte-mediated transport, all of which are vital for maintaining systemic metabolic balance [29,30]. Dietary rutin (250–500 mg/kg) can inhibit *Bax* or enhance *Ki67* expression to improve intestinal morphology and enhance intestinal nutrition absorption in broilers and weaned piglets [31,32]. Similarly, the results of our present study showed that dietary rutin at 300–1200 mg/kg reversed the HZn-induced upregulation of *Bax* and downregulation of *Ki67* mRNA expression in the jejunum. It is well known that *Bax* plays a critical role in the mitochondrial pathway of apoptosis by promoting the release of cytochrome c from mitochondria, which triggers caspase activation and ultimately leads to programmed cell death. In addition, *Ki67* is a proliferation-associated antigen whose function is closely related to mitosis, and it is indispensable for cell proliferation. These findings suggest that dietary rutin may effectively improve intestinal morphology and further enhance zinc absorption in mice treated with HZn. Sreenivasulu et al. [33] reported that quercetin, a compound rich in phenol groups and serving as the main intestinal metabolite of dietary rutin, can enhance metallothionein expression and stimulate zinc uptake in intestinal Caco-2 cells, which resonates with our previously reported results. Therefore, the reason for the unaffected or even higher zinc concentrations in tissues of mice from HZn + R1, HZn + R2, and HZn + R3 groups may be due to the enhanced intestinal zinc absorption by dietary rutin via improving intestinal morphology or enhancing expression of zinc absorption-related transporters [33,34].

Serum GOT and AKP activity, along with hepatic structural integrity, serve as critical indicators of liver injury [35,36]. In the present study, the HZn group significantly enhanced serum GOT and AKP activities in mice. In addition, the hepatic tissues of mice in the HZn group showed obvious cell lysis, disarrangement, and increased cellular gap, suggesting that dietary exposure to HZn may induce severe liver injury. Similarly, high nano-ZnO treatment (dietary 5000 mg/kg or gastrointestinal administration with 250 mg/kg body weight nano-ZnO) could lead to liver injury in mice [6,7]. However, in the current study, no significant differences in serum GPT, GOT, and AKP activities were observed between the control group and the 300–1200 mg/kg rutin groups. Dietary rutin also attenuated the HZn-induced cellular damage (reduced cellular gap and lysis), suggesting that dietary rutin could partially reverse HZn-induced liver injury in mice.

Additionally, the present study demonstrated that the HZn + R2 group could partially reverse HZn-induced hepatic injury in mice. Notably, this group also alleviated HZn-induced intestinal damage, as evidenced by inhibited *Ki67* expression and upregulated *Bax* expression; reduced organelle lysis, vacuolated mitochondria, and decreased expression of *Mfn2* and *Dnm1l*. The *Dnm1l* gene encodes dynamin-related protein 1 (Drp1), which is a key mediator of mitochondrial fission. Drp1 is recruited from the cytosol to the outer mitochondrial membrane, where it oligomerizes, hydrolyzes guanosine triphosphate (GTP), and forms spirals around the mitochondria. This constricts both the outer and inner membranes to complete mitochondrial fission. Previous research shows that *Mfn2* deficiency exacerbates ROS production and mitochondrial DNA (mtDNA) damage in hepatocytes [37]. *Mfn2* is involved in the regulation of various physiological processes in liver cells. In acute or chronic liver failure, studies have found that *Mfn2* can significantly attenuate the symptoms of liver failure, improve the expression of autophagy related proteins, and downregulate the expression of apoptosis-related proteins through the PI3K/Akt/mTOR signaling pathway. In addition, *Mfn2* can also restrain the proliferation of hepatic stellate cells, inhibit liver fibrosis, and play a protective role in the liver [38]. The observed *Mfn2* downregulation in the HZn + R1 group likely reflects a compensatory feedback mechanism to eliminate damaged mitochondria via enhanced mitophagy rather than direct rutin-mediated inhibition. This dynamic modulation of mitochondrial quality control pathways suggests context-dependent roles of rutin in cellular stress adaptation under zinc overload conditions.

It has been suggested that the injury may be associated with HZn-induced antioxidant imbalance, which further triggers oxidative stress; damages lipids, proteins, and DNA; and impairs cellular structure and mitochondrial function [9,10,11,39]. The current study found that dietary HZn significantly reduced serum or jejunal T-SOD activity and T-AOC level and increased the jejunal MDA content, while the latter was effectively reduced by dietary 600 mg/kg rutin. As a key enzyme for scavenging reactive oxygen species (ROS), SOD efficiently converts the highly dangerous superoxide anion into hydrogen peroxide, which is subsequently decomposed into H₂O by catalase. This process plays critical roles in maintaining tissue function and human health [40,41]. T-AOC is an important estimator of total antioxidant capacity, while MDA is known as a hallmark of lipid peroxidation [42]. Therefore, our results suggest that dietary rutin could improve the decreased antioxidant capacity caused by HZn in mice. This is consistent with the beneficial effects of rutin on the HZn-induced tissue injury. *Nrf2* is a major translation factor to manage a set of genes, including SOD, and activate the antioxidant system [31,32]. Our current study suggests that dietary rutin may reverse the HZn-induced decrease in *Nrf2* and *Sod1* mRNA expression, which might help explain the enhanced antioxidant capacity observed with dietary rutin. This finding aligns with previous reports in mice, weaned piglets, and broilers, where dietary rutin has been shown to potentially enhance antioxidant capacity by activating the Nrf2 signaling pathway [31,32,43].

These findings carry significant translational implications given the increasing human exposure to engineered nano-ZnO through consumer products (e.g., sunscreens, textiles) and environmental contamination. As a naturally occurring flavonoid abundant in dietary sources such as buckwheat and citrus, rutin emerges as a promising nutraceutical candidate for alleviating nano-ZnO-associated toxicity in humans. However, interspecies metabolic disparities—including reduced bioavailability and slower flavonoid metabolism in humans relative to rodents—highlight the need for rigorous dose optimization and advanced formulation strategies (e.g., nanoencapsulation) to ensure therapeutic efficacy.

## 5. Conclusions

Dietary rutin (especially for 600 mg/kg) could partially restore hepatic function and reverse relative organ indexes decreases without directly inhibiting the high zinc concentration in serum and tissue induced by HZn. Moreover, rutin could alleviate HZn-induced hepatic and jejunal injury (increased cell apoptosis and autophagy and damaged mitochondrial structure and function), likely via activating the *Ki67* signal to enhance antioxidant capacity.

## Figures and Tables

**Table 1:** Primer sequences used in quantitative real-time PCR assays.

Gene	Accession No.	Sequence (5′ to 3′)	Size (bp)
*β-actin*	NM_007393	F: AGCCATGTACGTAGCCATCC	194
		R: CTCTCAGCTGTGGTGGTGAA	
*Bax*	NM_007527.3	F: TGCAGAGGATGATTGCTGAC	173
		R: GATCAGCTCGGGCACTTTAG	
*Caspase-3*	XM_017312543.3	F: GTGAGCCGGTGCGGTG	250
		R: ACATCCGTACCAGAGCGAGA	
*Ki67*	XM_006507413.5	F: CCATCATTGACCGCTCCTTT	145
		R: TCACTCTTGTCAGGGTCAGC	
*P53*	AB021961.1	F: AAGGATGCCCATGCTACAGAG	223
		R: GAGTGGATCCTGGGGATTGTGTC	
*Hsp90*	NM_008302.3	F: GCGGCAAAGACAAGAAAAAG	167
		R: CAAGTGGTCCTCCCAGTCAT	
*Hsp40*	AB028272.1	F: CCAATGGGTATGGGTGGCTT	81
		R: ATCTTGCTTCTTCCGGGTGG	
*Nrf2*	NM_010902	F: CTCGCTGGAAAAAGAAGTGG	240
		R: CCGTCCAGGAGTTCAGAGAG	
*Sod1*	NM_011434.2	F: CCAGTGCAGGACCTCATTTT	197
		R: TTGTTTCTCATGGACCACCA	
*Gpx1*	NM_008160	F: GTCCACCGTGTATGCCTTCT	152
		R: TCTGCAGATCGTTCATCTCG	
*Dnm1l*	NM_001360010.1	F: GCCTCAGATCGTCGTAGTGG	194
		R: TTTTCCATGTGGCAGGGTCA	
*Mfn2*	NM_001355590.1	F: CTCCATTCAAGAAGCTTGGACAG	229
		R: ACTTCAGCCATGTGTCGCTT	
*Fis1*	NM_001347504.1	F: TACTCCTTCTACCCCGAGGC	279
		R: TCCTTGCAGCTTCGTCTCTG	

*Bax*, BCL2-associated X protein; *Hsp90*, heat shock protein 90; *Hsp40*, heat shock protein 40; *Nrf2*, nuclear factor erythroid-2-related factor 2; *Sod1*, superoxide dismutase 1; *Gpx1*, glutathione peroxidase 1; *Dnm1l*, dynamin 1-like; *Mfn2*, mitofusin 2; *Fis1*, fission.

## Data Availability

The original contributions presented in this study are included in the article. Further inquiries can be directed to the corresponding author.

## Abbreviations

The following abbreviations are used in this manuscript:

MDA	malondialdehyde
T-SOD	total superoxide dismutase
GOT	glutamic oxalacetic transaminase
GPT	glutamic pyruvic transaminase
AKP	alkaline phosphatase
T-AOC	total antioxidant capacity
Bax	BCL2-associated X protein
Hsp90	heat shock protein 90
Hsp40	heat shock protein 40
Nrf2	nuclear factor erythroid-2-related factor 2
Sod1	superoxide dismutase 1
Gpx1	glutathione peroxidase 1
Dnm1l	dynamin 1-like
Mfn2	mitofusin 2
Fis1	fission, mitochondrial 1
